# Immunogenic cell death genes in single-cell and transcriptome analyses perspectives from a prognostic model of cervical cancer

**DOI:** 10.3389/fgene.2025.1532523

**Published:** 2025-04-07

**Authors:** Li Ning, Xiu Li, Yating Xu, Yu Si, Hongting Zhao, Qinling Ren

**Affiliations:** ^1^ Affiliated Hospital of Nanjing University of Chinese Medicine, Nanjing, China; ^2^ The Chinese Clinical Medicine Innovation Center of Obstetrics, Gynecology, and Reproduction in Jiangsu Province, Nanjing, Jiangsu, China

**Keywords:** multi-omics, single-cell RNA, ICDs, immune prognosis, therapy efficacy, algorithms

## Abstract

**Background:**

The role of immunogenic cell death (ICD) in cervical cancer (CESC) is not well understood. This study sought to investigate the significance of ICD in CESC and to establish an ICDRs prognostic model to improve immunotherapy efficacy for patients with cervical cancer.

**Methods:**

ICD-associated genes were screened at the single-cell and transcriptome levels based on AddModuleScore, single-sample gene set enrichment analysis (ssGSEA) and weighted gene co-expression network (WGCNA) analysis. Immunogenic cell death-related features (ICDRs) were constructed using multiple machine algorithms, and ICDRs were evaluated in training and validation sets to provide quantitative tools for predicting prognosis in clinical practice. Predictive models were used to risk subgroups for response to immunotherapy, as well as drug sensitivity. Finally, the expression of ICD-related genes was verified by RT-qPCR.

**Results:**

Through an integrated analysis of single-cell data, transcriptomic profiling, and computational modeling, seven ICD-related genes were identified as highly prognostic for CESC patients. Multivariate analysis demonstrated that low-risk patients had significantly better overall survival compared to high-risk patients, confirming the model as an independent prognostic tool. Assessments of the tumor microenvironment (TME), mutation characteristics, and drug sensitivity within ICDRs risk subgroups indicated a stronger immunotherapy response in the low-risk group.

## 1 Introduction

Cervical cancer is the fourth most common cancer worldwide, with approximately 660,000 new cases reported in 2022 and approximately 350,000 deaths (Bray et al., 2024). It remains a significant health issue, particularly in low- and middle-income countries, where it is a leading cause of cancer incidence and mortality (Girda et al., 2023). Although the World Health Organization (WHO) has launched a global strategy to eliminate cervical cancer, including recommending HPV vaccination for girls under 15, the decline in incidence has been limited (Malagón et al., 2024).

Modern treatments for cervical cancer include radiotherapy, chemotherapy, targeted therapy and immunotherapy (Li et al., 2023; Tang and Chen, 2024). From human papillomavirus (HPV) infection to cervical carcinogenesis involves a series of complex regulatory mechanisms, among which immunotherapy plays a crucial role in tumour development and progression and has become a key area of current research (Awadasseid et al., 2023). The application of immunotherapy in cervical cancer mainly includes strategies such as immune checkpoint inhibitors, therapeutic vaccines, and adoptive T-cell immunotherapy (Ferrall et al., 2021). By targeting immune checkpoints and modulating tumour immune escape mechanisms, immunotherapy shows promising therapeutic perspectives and has become an important direction in clinical applications and basic research (Fobian et al., 2025). Patients with advanced or recurrent cervical cancer benefit from reducing immunosuppression in the tumour microenvironment by targeting the PD-1/PD-L1 pathway, which is associated with T-cell exhaustion, and the CTLA-4 pathway, which inhibits T-cell activation (Horton et al., 2018; Santoro et al., 2024; Liu K. et al., 2024). However, many patients fail to benefit from this, suggesting that further research is urgently needed to explore reliable predictive biomarkers to identify high-risk patients and guide individualised treatment to improve prognosis.

Immune cell death (ICD) has been recognized as a promising therapeutic strategy as a regulated cell death process (Wang et al., 2018). ICD, as a regulated cell death, is capable of being induced by a variety of stimuli, including pathogen infection, chemotherapy, targeted drug therapy, and photodynamic therapy (Liu et al., 2024). By triggering the death of tumor or infected cells through external stimuli, ICD prompts the transformation of these cells from non-immunogenic to immunogenic, thereby enhancing anti-tumor immune responses and establishing long-term immune memory (Catanzaro et al., 2025). This process typically relies on the release of immunogenic molecules (DAMPs), which in turn activate adaptive immune responses (Wang et al., 2018; Fan et al., 2024). DAMPs enhance the immunogenicity of tumor cells, attenuate immunosuppression in the tumor microenvironment, stimulate T-cell-mediated immune responses, and ultimately promote tumor-specific CD8^+^ T cell generation and the establishment of immune memory (Widjaya et al., 2022). Currently, ICD-based immunotherapy has become an important direction in tumor therapy. Both chemotherapy (Yerragopu and Vellapandian, 2023), radiotherapy (Pointer et al., 2022), photoimmunotherapy (Li et al., 2020), and tumor vaccines can induce ICD and improve the therapeutic effect (Wang et al., 2024b). However, the potential of ICD as a prognostic biomarker or predictor of response to immunotherapy and chemotherapy has not been fully explored, and further research strategies are still needed especially in cervical cancer patients. Therefore, studying and identifying reliable ICD biomarkers is crucial for assessing the prognosis and treatment response of cervical cancer patients. A large number of studies have explored new therapeutic targets by constructing survival prediction models, a trend that highlights the important potential of this field in tumor therapy (Zhang et al., 2024c; Zhang et al., 2024d; Zhang et al., 2024a; Zhang et al., 2024b).

In this study, we constructed a prediction model for ICDRs associated with CESC based on 33 known ICD-related genes using a multi-omics analysis combined with various machine learning algorithms. We divided cervical cancer patients into two subtypes and verified significant differences between the subtypes in terms of clinical characteristics, prognosis, gene mutations, tumour microenvironment (TME), immune checkpoint expression and drug sensitivity, thus providing new ideas for predicting the progression of cervical cancer.

## 2 Material and methods

### 2.1 Data preparation

Data on cervical cancer, including transcriptomic, mutational, and clinical information, were sourced from the TCGA and GEO databases. Clinical data came from TCGA, and TPM values were extracted for analysis. Genes with an average expression below 0.1 and samples lacking complete clinicopathological data were excluded, forming the TCGA-CESC cohort. Single-cell RNA sequencing data were obtained from the GSE44001 dataset. Additional somatic mutation data in Mutation Annotation Format (MAF) were obtained from The Cancer Genome Atlas (TCGA), while copy number variation (CNV) data specific to TCGA-CESC patients were retrieved from the Xena database. The IMvigor210 cohort, consisting of patients who received immune checkpoint blockade (ICB) therapy, was utilized to evaluate the efficacy of the ICDRs model in predicting sensitivity to immunotherapy (Mariathasan et al., 2018). A set of 34 ICD-related genes (Garg et al., 2016; Wang et al., 2021), identified from previous studies, is presented in Supplementary Table S1.

### 2.2 Single-cell data processing

Single-cell RNA sequencing analysis and data processing were conducted using the Seurat package (Stuart et al., 2019). Quality control procedures were implemented to exclude genes expressed in fewer than 3 cells and cells expressing fewer than 200 genes. Cells expressing between 200 and 4,000 genes, with mitochondrial gene content below 10%, were retained for further analysis. Mitochondrial and ribosomal RNA (rRNA) ratios were quantified utilizing the Percentage FeatureSet function. To reduce data dimensionality, principal component analysis (PCA) was conducted, with 20 principal components selected as anchors (dim = 20). Batch effects across samples were addressed using the Harmony package. Subsequently, dimensionality reduction was achieved through the application of the t-distributed stochastic neighbor embedding (t-SNE) function. Dimensionality reduction was performed using the t-SNE algorithm. Following this, cell subpopulations were identified through the application of the FindNeighbors and FindClusters functions, with a resolution parameter set at 0.1. The AddModuleScore function from the Seurat package was utilized to evaluate the activity of specific gene sets across individual cells. Differentially expressed genes (DEGs) between groups were identified using the FindMarkers function, with statistical significance determined via the Wilcoxon test and an adjusted p-value threshold of less than 0.05. Differentially expressed genes (DEGs) identified between cells exhibiting high and low immunogenic cell death (ICD) scores at the single-cell transcriptomic level were classified as ICD-related. These genes were subsequently integrated into a weighted gene co-expression network analysis (WGCNA). Additionally, the R package CellChat was utilized to investigate intercellular communication (Jin et al., 2021).

### 2.3 WGCNA batch identification of immunogenicity-related genes

Weighted Correlation Network Analysis (WGCNA) is utilized as a systems biology methodology to identify patterns of association among samples (Langfelder and Horvath, 2008). Through WGCNA, modules and genes exhibiting the strongest correlation with immunogenic cell death-related genes (ICDRs) are identified. After clustering the samples and eliminating outliers, an appropriate soft threshold is selected to ensure optimal performance and model stability. Subsequently, differentially expressed genes are intersected with those identified by WGCNA, representing ICDRs.

### 2.4 Development and validation of prognostic indicators related to ICDRs

To analyze prognostic features associated with ICDR, RNA sequencing data from the TCGA database was utilized. Differentially expressed genes between normal and tumor samples were identified using the R package limma, with thresholds of |logFC| > 0.5 and adjusted p-value <0.05. Genes overlapping between the differentially expressed genes and ICD-related modules from weighted gene co-expression network analysis (WGCNA) were selected as ICDR. Patients were stratified into high- and low-risk groups based on the median ICDR risk score. Kaplan-Meier curves were generated for prognostic analysis, and statistical significance was evaluated using the chi-square test. Univariate and multivariate analyses were conducted to assess the combined effects of clinicopathological factors on survival. Time-dependent ROC curves for predicting 1-, 3-, and 5-year survival rates were constructed, and AUC values were calculated to assess model accuracy. A nomogram was created using the R package RMS, and the relationship between risk scores and clinical factors, including age and TNM stage, was analyzed. The accuracy and reliability of the nomogram were evaluated with ROC curves and calibration plots, while decision curve analysis (DCA) was used to assess its net clinical benefit.

### 2.5 Gene set enrichment analysis

Single-sample gene set enrichment analysis (ssGSEA) is a prevalent technique utilized to evaluate the enrichment score of specific gene sets within individual samples. The ssGSEA score for each sample indicates the extent of upregulation or downregulation of a particular gene set in that sample. To discern differentially expressed genes and their variations between high- and low-risk ICDR groups, we utilized the R package “limma.” Furthermore, functional enrichment and differences in biological pathways between these groups were examined using the “org.Hs.eg.db” and “clusterProfiler” packages. Gene set files for GO terms, KEGG pathways, and HALLMARK pathways in GMT format were obtained from MsigDB (version 4.0) (Castanza et al., 2023).

### 2.6 Correlation analysis between immune infiltrating cells and gene mutations

To investigate the immunological significance of ICDRs, we used the CIBERSORT, ESTIMATE and ssGSEA algorithms. Immunity, stroma, ESTIMATE score and tumour purity were calculated using the ESTIMATE algorithm. In addition, activity scores for the seven steps of the anti-tumour immune cycle were assessed. Immunotherapy responses between high and low risk groups were analysed using the Immunophenotype Scoring (IPS) algorithm, and IPS data for the TCGA-CESC samples were obtained from the Cancer Immunome Atlas (TCIA) database (https://tcia.at/home) (Yu et al., 2023).

Given the association between increased genetic heterogeneity of tumours and poor prognosis in cancers such as head and neck squamous cell carcinoma and breast cancer, as indicated by the Mutant Allele Tumour Heterogeneity (MATH) score, MATH was used in this study to measure tumour heterogeneity (Mroz et al., 2013; Mroz et al., 2015). The mutation profiles of CESC patients were analysed using the maftools R software package and gene copy number variations (CNVs) were determined for genes that differed between the two risk groups.

### 2.7 Significance of the ICDRs in drug sensitivity

We evaluated the half maximum inhibitory concentration (IC50) of common clinical chemotherapy and targeted drugs by using “pRRophetic” software package. We used Wilcoxon test to check the difference of IC50 between high and low risk groups, and p < 0.05 was considered to be statistically significant.

### 2.8 Experimental validation of key ICDRs expression via RT-qPCR in CESC

RNA was extracted from two cervical cancer cell lines (SiHa and Hela) and the immortalized epithelial cell line HaCaT using the RNA extraction kit (R0027; Beyotime Biotechnology, Nanjing, China). The RNA was reverse-transcribed into cDNA using the PrimeScript RT reagent kit (R323-01; Vazyme). Real-time PCR (RT-PCR) was conducted on a QuantStudio five system, and data were analyzed based on comparative CT values. Gene expression levels were normalized to GAPDH as a reference. Results are presented as mean ± standard deviation (SD) from three independent experiments. Primer sequences are provided in Supplementary Table S6.

### 2.9 Statistical analyses

All statistical analyzes were performed using R software (version 4.4.1). Differences between groups were analyzed using the Wilcoxon test, and KM curves were analyzed using the Log-rank test. Univariate and multivariate Cox regression were performed for independent prognostic analysis. Spearman’s correlation analysis was used to examine the relationship between risk score and immune cell infiltration. RT-qPCR results were analyzed using the student’s test. Two-sided P values less than 0.05 were considered statistically significant.

## 3 Result

### 3.1 Analysis of immune cell death genes in single-cell transcriptome data

**FIGURE 1:**
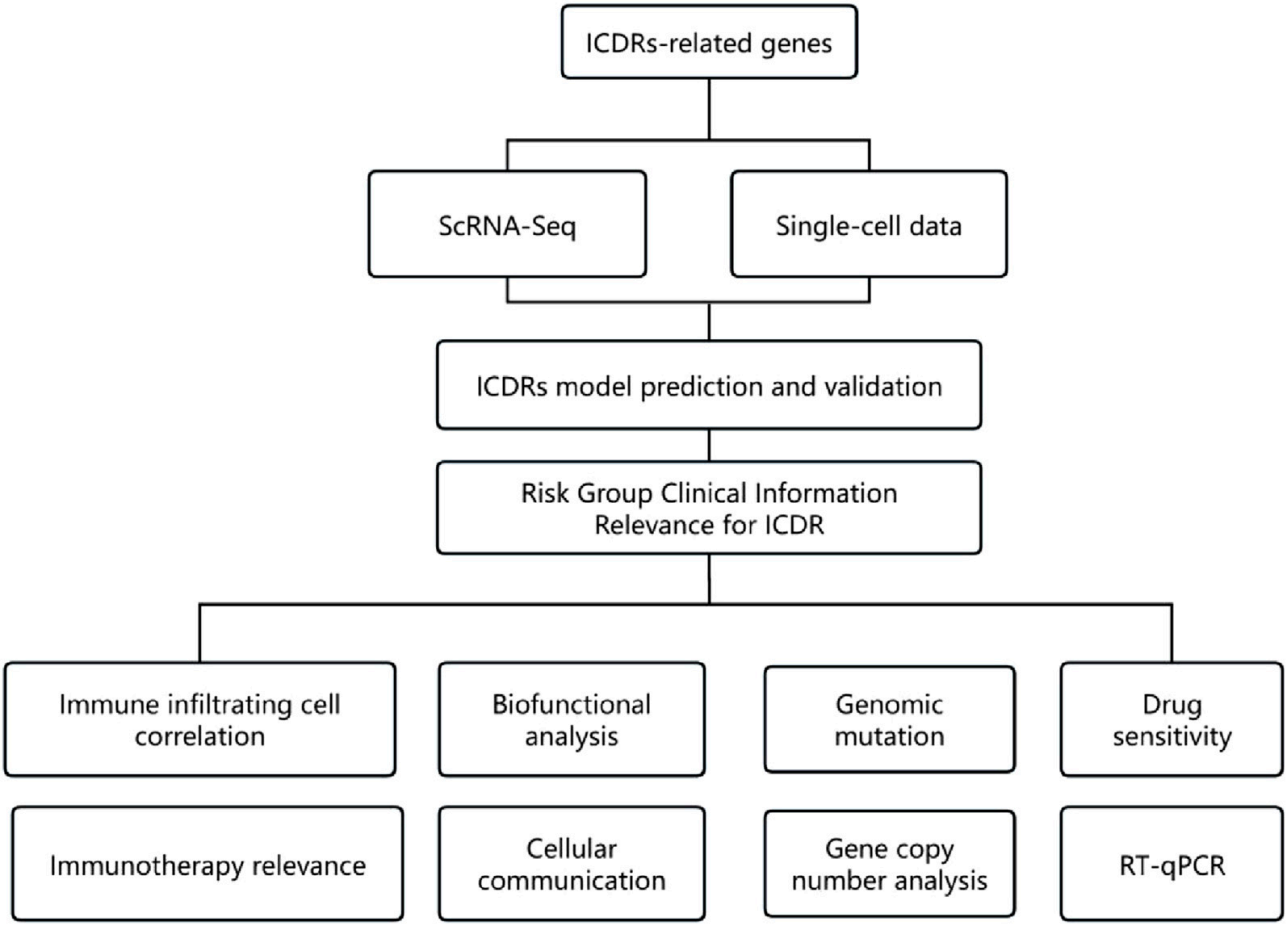
Flowchart in this study.

**FIGURE 2:**
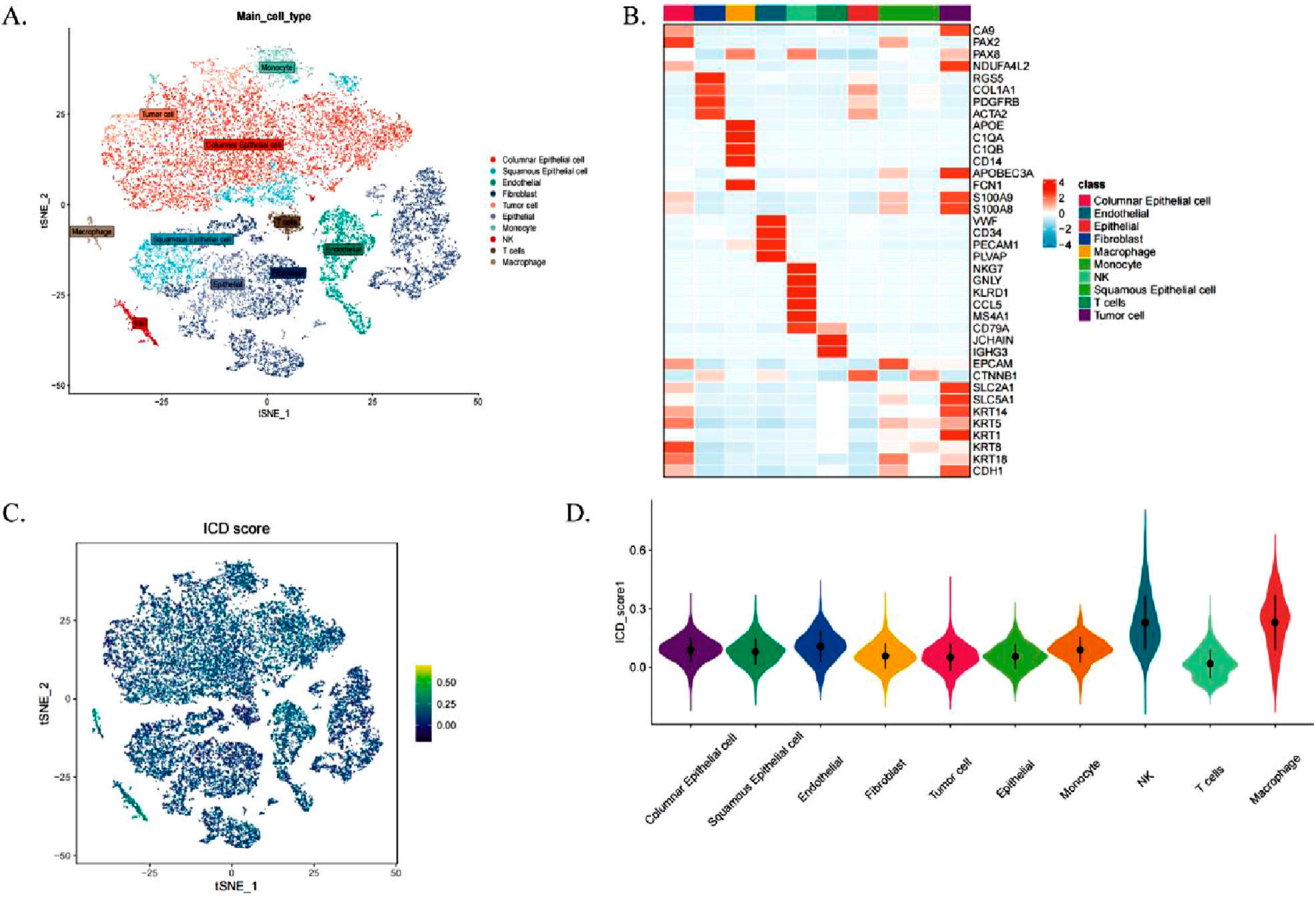
Single Cell Transcriptome Analysis of Immunogenic Cell Death (ICD) Gene. **(A)** t-SNE plot illustrating cell types identified based on marker genes. **(B)** Heatmap displaying the top four marker genes for each cell population. **(C)** Immunogenic cell death (ICD) activity scores across individual cells. **(D)** Distribution of ICD scores among various cell types.

Figure 1 outlines the study’s workflow. We analysed the scRNA-seq dataset GSE44001 and identified 24,302 cells divided into 31 cell clusters (Supplementary Figure S1A). Based on marker genes, these clusters were further classified into 10 cell types, including fibroblasts, macrophages, monocytes, endothelial cells, NK cells, T cells, epithelial cells, columnar epithelial cells, squamous epithelial cells and tumour cells (Figure 2A). The heatmap shows the top four marker genes for each cell type (Figure 2B). To assess the activity of immunogenic cell death (ICD) genes in different cell types, we calculated the expression levels of 34 ICD-related genes in individual cells using the ‘AddModuleScore’ function in Seurat (Figure 2C). Among the 10 cell types, NK cells, macrophages and endothelial cells had the highest ICD activity (Figure 2D). Subsequently, the cells were classified into high and low ICD groups based on their ICD activity, and 349 differentially expressed genes were identified for further analysis (Supplementary Table S2).

### 3.2 Identification of modular genes related to ICDs through WGCNA analysis using large-scale transcriptomic data

ICD as an anti-cancer therapeutic ‘saviour’ capable of activating adaptive immune responses (Galluzzi et al., 2024). We aimed to derive a gene set enrichment score using the ssGSEA algorithm for specific samples to identify differences in pathway activity or biological functions across samples. ICD gene activity scores were calculated for each TCGA-CESC sample by using the ssGSEA algorithm, and these scores were used as phenotypic data in the WGCNA analysis.

**FIGURE 3:**
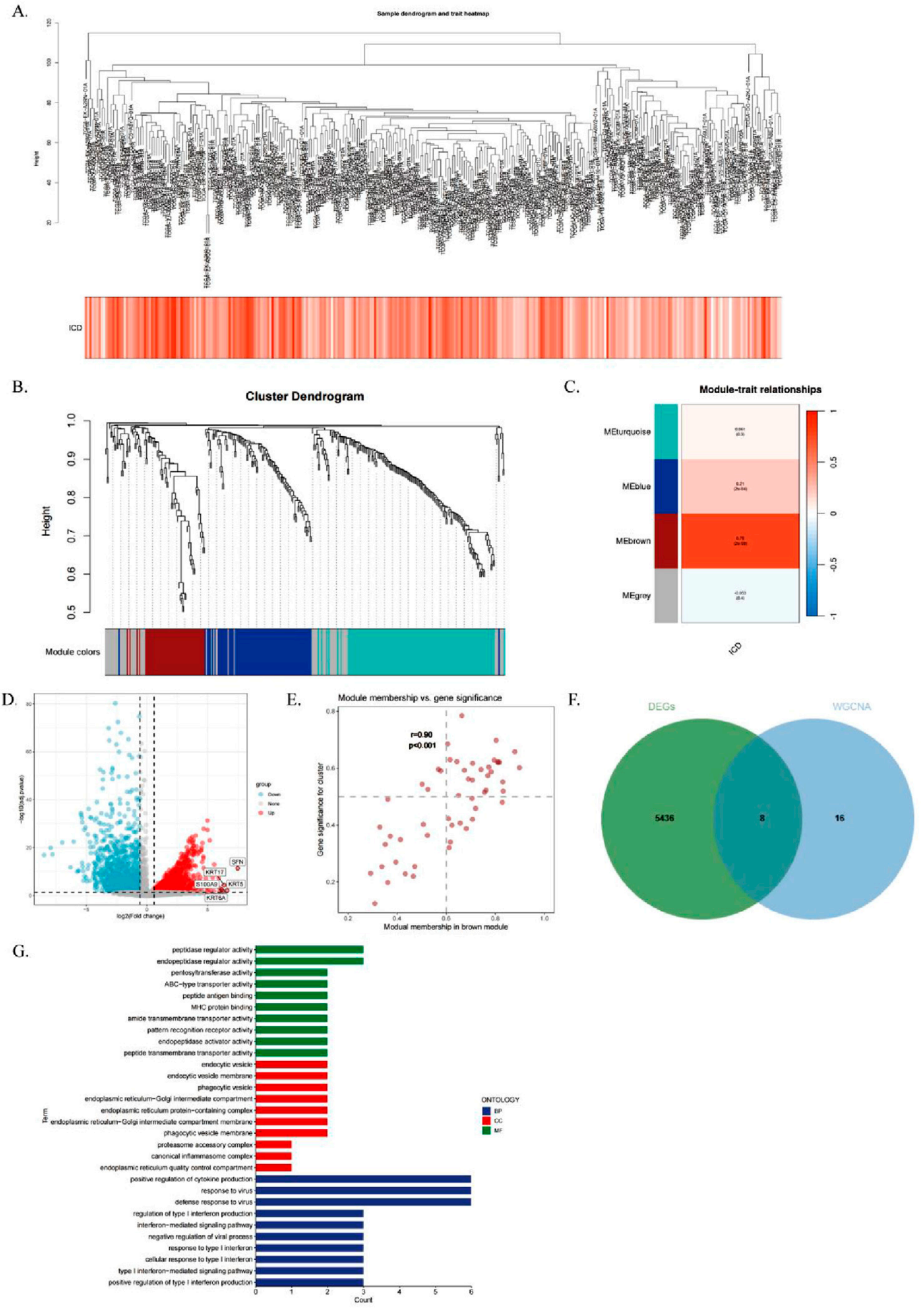
Single Cell Transcriptome Analysis of Immunogenic Cell Death (ICD) Gene. **(A)** Hierarchical clustering dendrogram of TCGA-CESC samples, with a heatmap below showing ICD scores calculated via the ssGSEA algorithm. **(B)** WGCNA clustering dendrogram analysis. **(C)** Module-trait relationships. Heatmap showing correlations between genetic modules and clinical features, with red indicating positive correlations and blue indicating negative correlations. p-values are shown in parentheses. **(D)** Scatterplot depicting the correlation between gene significance (GS) and module membership (MM) in the brown module. **(E)** Volcano plot of differential analysis results for TCGA-CESC tumor and normal samples, with the top five up- and downregulated genes labeled. **(F)** Venn diagram illustrating overlapping genes between the MEbrown module and RNA-seq DEGs. **(G)** GO enrichment analysis of the identified genes.

To identify modules significantly associated with ICD gene scores, we conducted WGCNA on the TCGA-CESC dataset. After excluding outlier samples, co-expression networks were constructed using ICD-associated DEGs identified from single-cell sequencing data (Figure 3A). The optimal soft threshold power was set at 7 (R^2^ = 0.76) to ensure a scale-free topological network (Supplementary Figure S1B). Parameters included a minimum module gene number of 60 and a gene similarity threshold (MEDissThres) of 0.25, resulting in 18 distinct modules (Figure 3B).

The MEbrown module showed a strong correlation with ICD gene scores from RNA-seq data (correlation = 0.76, Figure 3C). Additionally, a significant positive correlation was observed between gene importance and module membership in the MEbrown module (correlation = 0.9, P < 0.001, Figure 3D). This indicates that the module not only exhibits structural consistency (co-expression patterns) but also contains core genes that play critical roles in ICD-related processes.

The differential gene expression analysis of cervical cancer samples and normal samples using a volcano plot (|logFC| > 0.5, adjusted P < 0.05; Figure 3E) showed differentially expressed genes (DEGs). By intersecting the 24 genes in the brown module with the RNA-seq DEGs, we identified eight genes to construct the risk score model (Figure 3F), which were termed immunogenic cell death-related genes (ICDRs).

Gene ontology (GO) enrichment analysis of the ICDR genes (Figure 3G) showed that these genes were involved in biological processes (BPs) such as ubiquitin-like protein ligase binding, endopeptidase regulator activity, pentosyltransferase activity, and MHC protein binding. In terms of cellular components (CC), these genes are associated with cytoplasmic vesicle lumen and secretory granule lumen. In terms of molecular function (MF), the ICDR genes are associated with pathways such as negative regulation of defence responses, suppression of immune responses, cellular responses to ICD and responses to type I interferon (Supplementary Table S3).

### 3.3 ICDs risk modeling predicts the prognosis of patients with CESC

**FIGURE 4:**
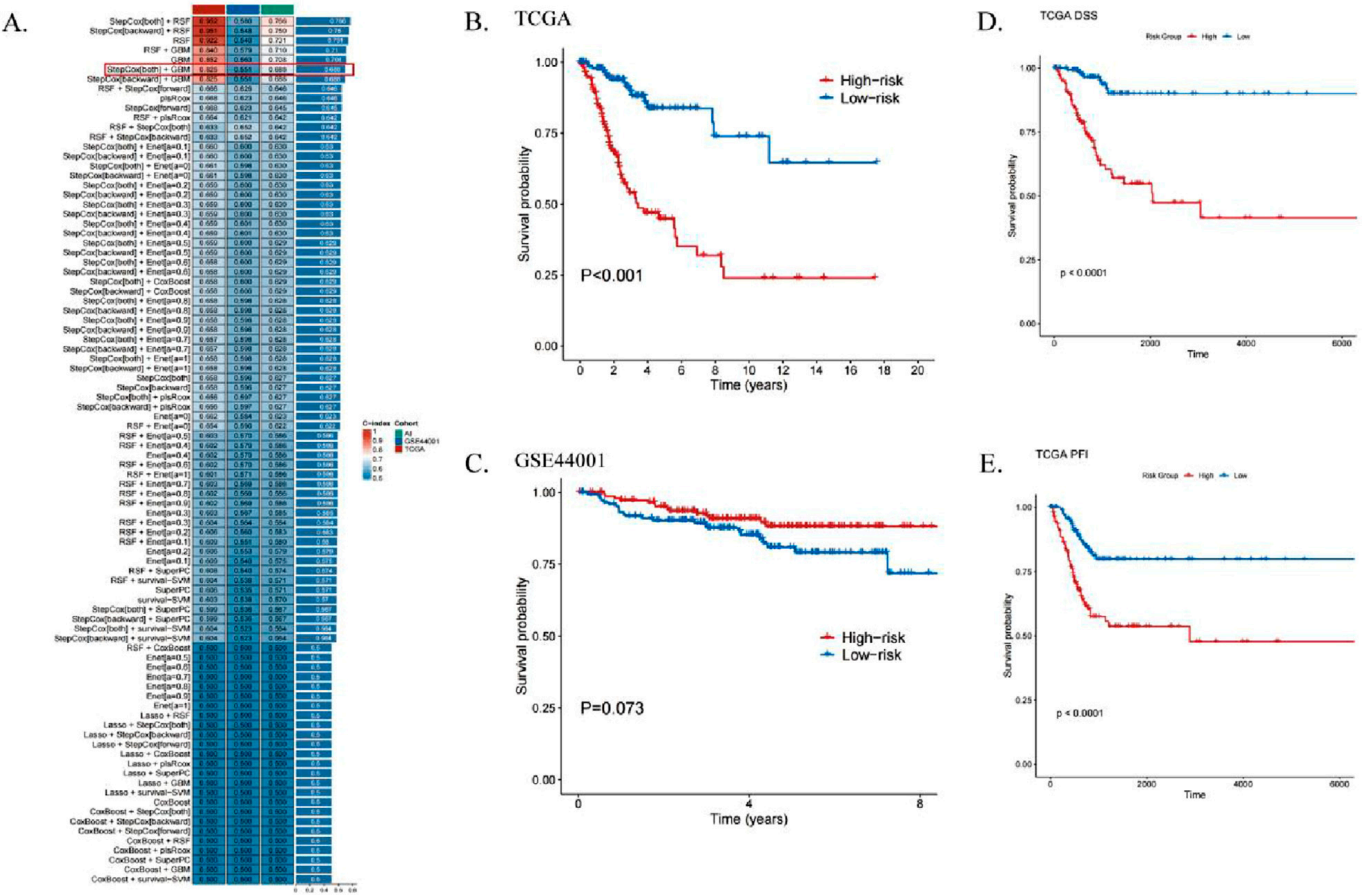
ICDRs risk modeling predicts the prognosis of patients with CESC. **(A)** Development of machine learning-based prognostic models for risk assessment, with the C-index calculated across all validation datasets. **(B, C)** Kaplan-Meier curves illustrating OS analyses for ICDR-based risk subgroups in the training set (TCGA) **(B)** and validation set (GEO) **(C)** using the log-rank test. **(D, E)** Kaplan-Meier curves showing DSS **(D)** and PFI **(E)** analyses within TCGA risk subgroups.

Univariate regression analysis identified six ICDRs significantly associated with CESC patient prognosis. The TCGA dataset was divided into a training set, while the GEO dataset was used as a validation set. Using 101 machine learning algorithms, predictive models were constructed, and their performance was evaluated by calculating the consistency index (C-index) for each validation cohort (Figure 4A). Among the algorithms, the StepCox algorithm performed well, but after excluding overfitting models in the training set, the StepCox [both]+GBM model demonstrated the best validity with an average C-index of 0.688.

KM survival analysis revealed that high-risk patients had significantly worse prognoses in the TCGA training set (Figure 4B). However, in the GEO dataset, although high-risk patients showed a similar trend, the p-value exceeded 0.05, indicating no significant difference (Figure 4C). Additionally, worse disease-specific survival (DSS) and progression-free interval (PFI) were observed in the low-risk group compared to the high-risk group (p < 0.001, Figures 4D, E).

### 3.4 Assess the independent prognostic significance of ICDs

**FIGURE 5:**
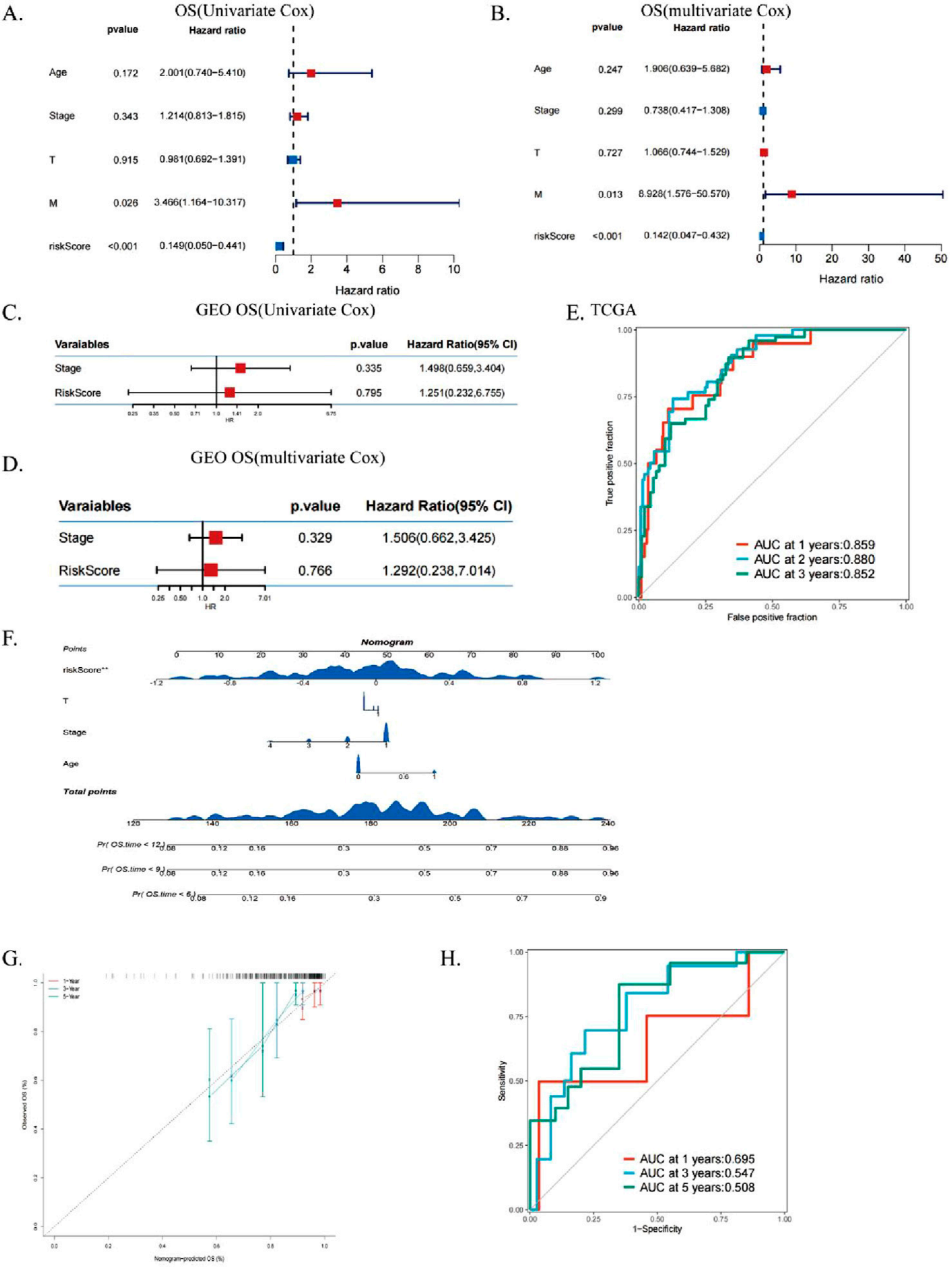
Assess the independent prognostic significance of ICDs. **(A, B)** Forest plots for univariate and multivariate prognostic analysis in TCGA-CESC. **(C, D)** Univariate and multivariate analysis of OS-related clinical characteristics and ICDRs in GEO. **(E)** ROC curves evaluating TCGA predictive performance for 1-, 3-, and 5-year OS. **(F)** Nomograms integrating ICDRs with clinical factors such as age, grade, stage, and T. **(G)** ROC curves assessing nomogram performance for predicting 1-, 3-, and 5-year OS. **(H)** Calibration curves of the nomogram for 1-, 3-, and 5-year OS predictions.

We performed univariate (Figure 5A) and multivariate (Figure 5B) Cox regression analysis to evaluate the role of ICDRs as an independent prognostic factor for CESC. The results confirmed that ICDRs significantly affected the prognosis of CESC and was independent of other clinical variables, so we used it as an independent prognostic indicator (P < 0.001), and the OS prognostic analysis of the validation cohort (Figures 5C,D) further confirmed the independent prognostic value of ICDR (HR 1.292, CI 0.238–7.014, P = 0.766), and DSS, DFI and PFI were similarly analyzed (Supplementary Figures S2A–C). In addition, ROC curve analysis showed that the AUC values at 1, 2, and 3 years were all high (0.859, 0.88, and 0.852, respectively), indicating that the model had high predictive reliability (Figure 5E). In order to verify the clinical applicability of the dicing model, the ICDR was combined with independent prognostic indicators to construct a prediction nomogram to quantitatively evaluate survival, age, and TNM stage (Figure 5F). The calibration curve verified the accuracy of the nomogram (Figure 5G). The calibrated AUC values at 1, 3, and 5 years were 0.695, 0.547, and 0.508, respectively (Figure 5H), reflecting the predictive ability of the prediction model over time.

### 3.5 Clinical relevance of predictive models for ICDRs

**FIGURE 6:**
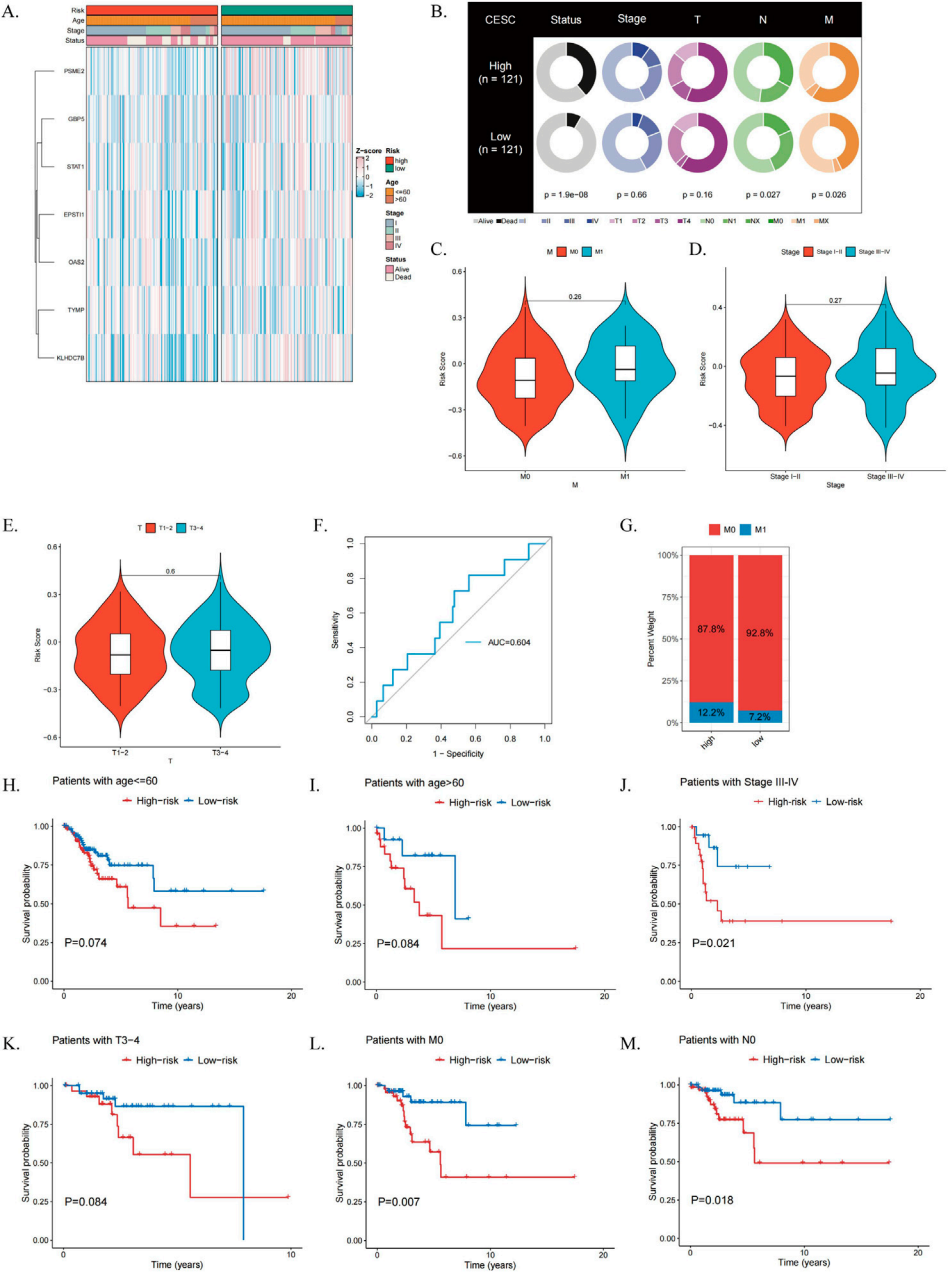
Evaluation of the Clinical Relevance of the ICDRs Risk Prediction Model. **(A)** Comparison of clinical characteristics of ICDR risk subgroups. **(B)** Correlation analysis of clinical characteristics between high-risk and low-risk subgroups. **(C–E)** Differences in risk scores between patients grouped by M stage, grading, and T stage. **(F)** Distribution of M stage in ICDR risk subgroups. **(G)** ROC curves assessing the predictive accuracy of ICDR for M-stage in CESC. **(H–M)** Kaplan-Meier survival curves for ICDR in patients with CESC by age, stage, T, M and N.

We conducted a correlation analysis between the clinicopathological characteristics of CESC patients and the ICDRs prediction model. The results from the TCGA dataset revealed a significant association between the risk score and several factors, including age, stage, and clinical status (Figure 6A). Furthermore, clinical Circos plot analysis demonstrated a statistically significant relationship between survival status and patient prognosis (P < 0.001), underscoring the critical role of survival status in tumor progression. Notably, a significant correlation was observed between survival status and N stage (Figure 6B). Compared with patients in early stages such as M0, I-II, and T1-2, patients in advanced stages, including M1 (P = 0.26), III-IV (P = 0.27), and T3-4 (P = 0.6), had significantly higher risk scores (Figures 6C–E). These findings suggest that ICDR is associated with a worse prognosis in patients with CESC. In addition, ROC curve analysis showed that ICDR achieved AUC 0.604 in predicting the M stage of CESC patients (Figures 6F, G). It is worth noting that the T4 stage distribution in the low-risk group was slightly higher than that in the high-risk group (Supplementary Figure S2M). These results indicate that combining ICDR with clinical information can improve the predictive accuracy of the model.

Furthermore, Kaplan-Meier survival analysis demonstrated that ICDRs have a distinct prognostic effect across various clinical subgroups, including age, stage, T, M, and N (Figures 6H–M; Supplementary Figures S2D–G). Supplementary analysis of five ICDRs-related genes was conducted using the GEPIA2 database (http://gepia2.cancer-pku.cn/) (Supplementary Figures S2H, I).

### 3.6 Biological functional differences between high and low risks

**FIGURE 7:**
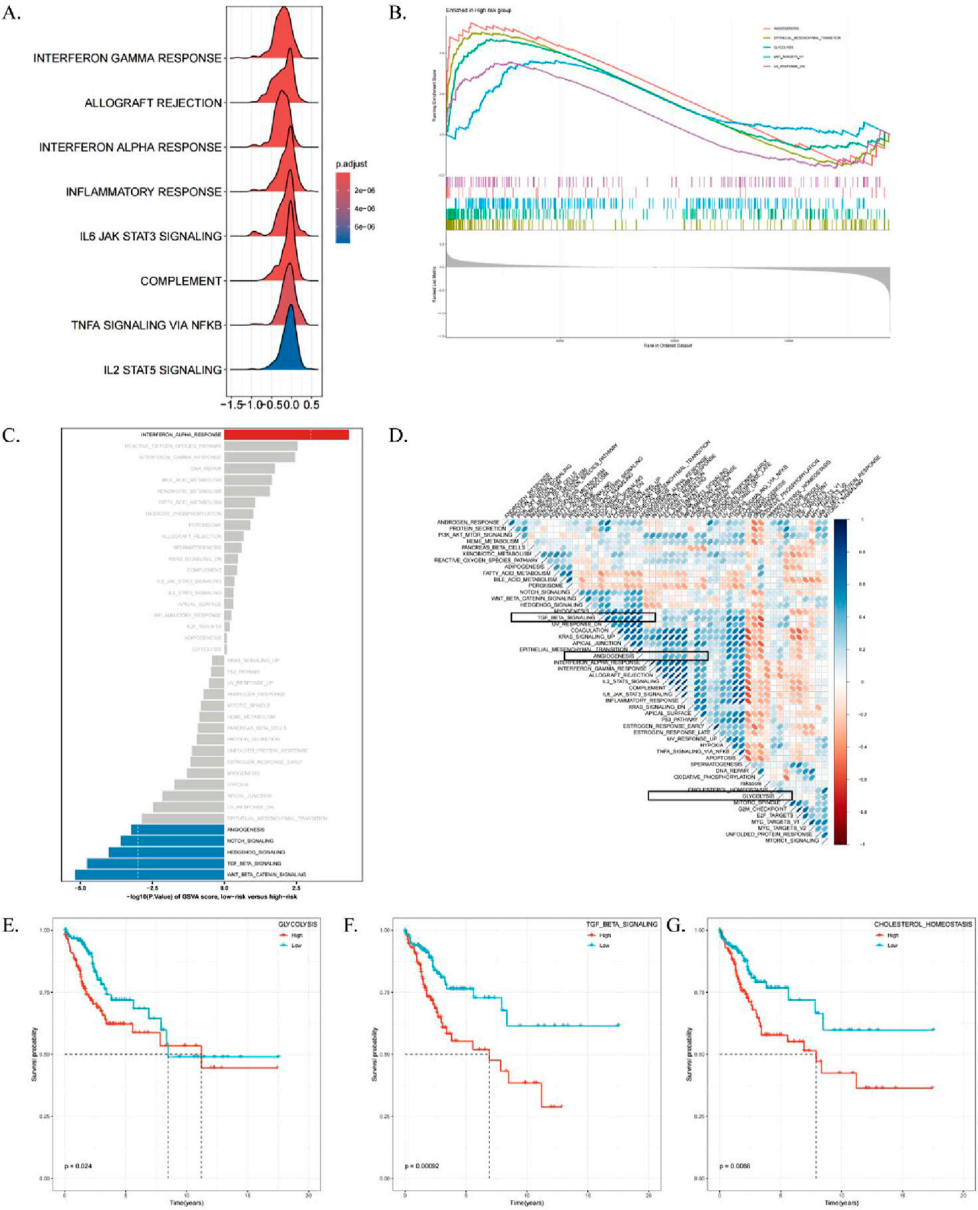
High-risk/low-risk functional enrichment analysis. **(A)** Mountain range plot displaying GO terms enriched in pathways specific to the low-risk group. **(B)** KEGG pathways enriched in the high-risk group. **(C)** Differential analysis of HALLMARK pathway activities between high- and low-risk groups based on GSVA scores. **(D)** Correlation analysis between GSVA scores of marker pathways and risk scores. **(E–G)** Kaplan-Meier curves showing the relationship between OS and GSVA scores for Glycolysis, TGF_BETA_SIGNALING, and CHOLESTEROL_HOMEOSTASIS pathways.

Given that ICDRs have a significant impact on the prognosis of CESC, we performed GSEA enrichment analysis on the risk subgroups of ICDRs to understand the differences in biological functions between different risk subgroups. We found that immune-related pathways such as INTERFERON_GAMMA_RESPONSE and ALLOGRAFT_REJECTION, cytokine signaling-related pathways such as IL6-JAK-STAT3 signaling pathway, and inflammatory response pathways such as type I interferon-α pathway were significantly enriched in the low-risk group (Figure 7A). In contrast, pathways related to EMT, glycolysis, angiogenesis, and MYC target gene regulation were significantly enriched in the high-risk group (Figure 7B). Further study findings (Supplementary Table S4) showed that the activities of glycolysis and Hedgehog signaling pathways were enhanced in the high-risk group, while the activities of ALLOGRAFT_REJECTION, INTERFERON_GAMMA_RESPONSE, IL6_JAK_STAT3, and IL2_STAT5 signaling pathways were significantly enhanced in the low-risk group (Figure 7C). Correlation analysis between ICDRs risk subgroups and other pathways (Figure 7D) showed significant associations with tumor-related pathways. The KM curve of the Hallmark pathway showed that pathways positively correlated with ICDR, such as glycolysis, TGF_BETA_SIGNALING, and CHOLESTEROL_HOMEOSTASIS, were associated with poor prognosis (Figures 7E–G). In contrast, KRAS_SIGNALING_DN and ALLOGRAFT_REJECTION was negatively correlated with the ICRD risk subgroup, which had a good prognosis. Moreover, IL-6_JAK_STAT3_SIGNALING, INFLAMMATORY_RESPONSE, P53_PATHWAY, and IL2_STAT5_SIGNALING showed better prognosis in the low-risk group (Supplementary Figures S3A–F). Collectively, these findings revealed significant differences in ICDRs risk subgroups with GO, KEGG, and Hallmark pathways, highlighting their potential as prognostic markers.

### 3.7 Tumor heterogeneity and mutation profiles across high-risk and low-risk groups

**FIGURE 8:**
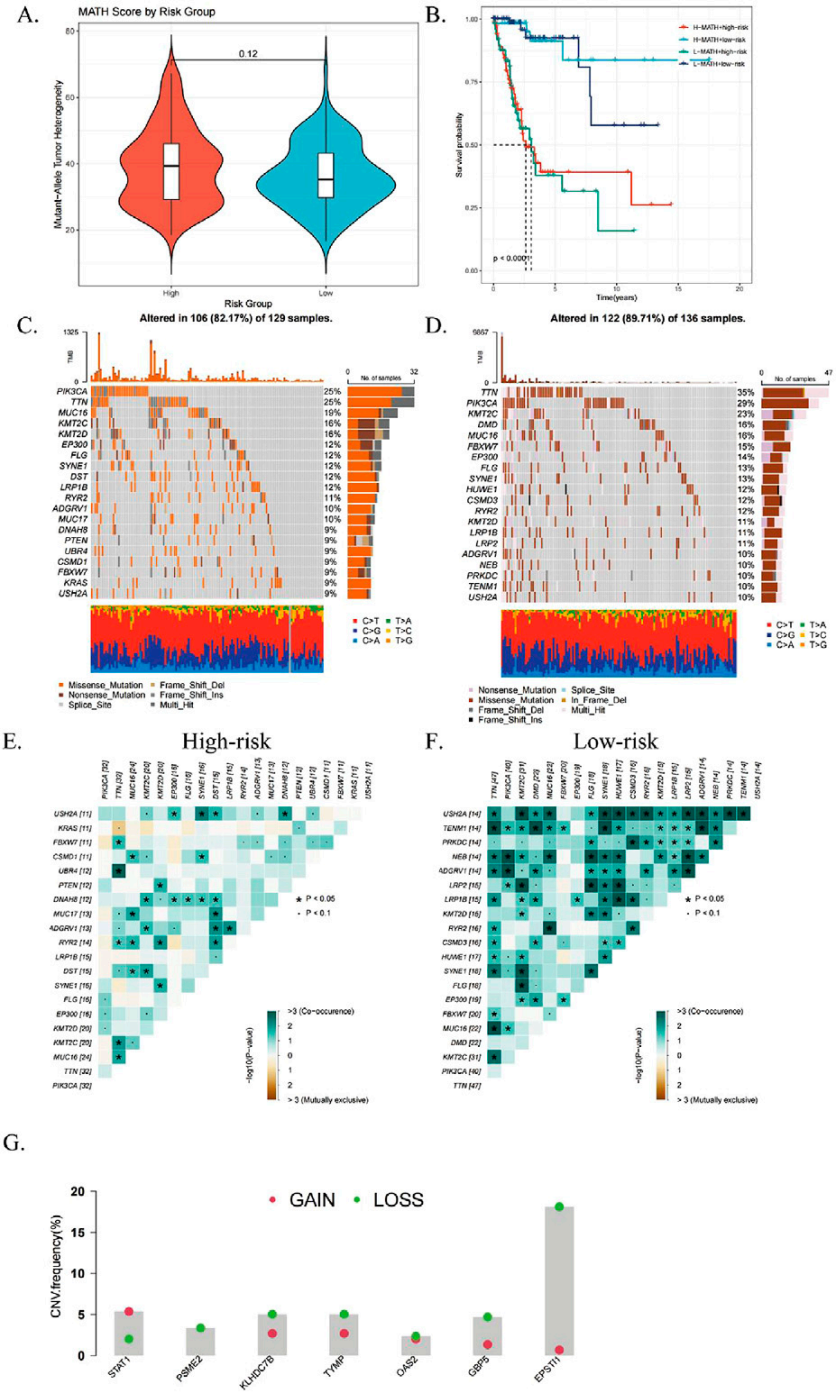
Relationship with genomic mutations and intratumor heterogeneity. **(A)** Violin plot comparing tumor heterogeneity (MATH) scores for mutant alleles between high- and low-risk subgroups. **(B)** Kaplan-Meier curves showing overall survival (OS) based on combined MATH and ICDRS risk scores. **(C, D)** Waterfall plots illustrating somatic mutation patterns in high- and low-risk subgroups. **(E, F)** Heatmaps displaying correlations of the top 20 mutated genes in high- and low-risk subgroups. **(G)** CNV variant levels in differential genes between subgroups, with red indicating amplifications and green deletions. *P < 0.05; **P < 0.01; ***P:< 0.001; ****P < 0.0001.

Tumor cells develop intratumoral heterogeneity (ITH) through continuous clonal evolution, which is closely associated with poor drug prognosis (Andrade et al., 2023). ITH results in the emergence of tumor cell subpopulations with higher proliferation rates, greater invasiveness, and varying drug sensitivities, complicating treatment strategies (Elza et al., 2014). ITH was quantified using the Mutated Allele Tumor Heterogeneity (MATH) algorithm, where higher MATH scores indicate greater heterogeneity. High-risk CESC patients exhibited significantly higher MATH scores compared to the low-risk group (Figure 8A).

The ICDRs prediction model and MATH were combined for prognostic evaluation, and the results showed that the prognosis of patients with “high risk + high MATH” was significantly worse than that of patients with “low risk + low MATH” (p < 0.0001, Figure 8B). This indicates that the combined index provides a more accurate prognosis for CESC patients. Further mutation analysis found significant differences in mutation rates (Figures 8C, D), with ADGRV1 and LRP1B mutations more frequently in the high-risk group. Fisher’s test confirmed that the mutation frequency of LRP1B was higher in the high-risk group, showing a co-mutation pattern (Figures 8E, F). In addition, we analyzed the copy number variation levels of the seven key genes of the ICDRs prediction model and found that except for the two genes STAT1 and KLHDC7B, the CNVs of other genes were significantly missing (Figure 8G).

### 3.8 Analysis of cellular communication between ICDR risk subgroups

**FIGURE 9:**
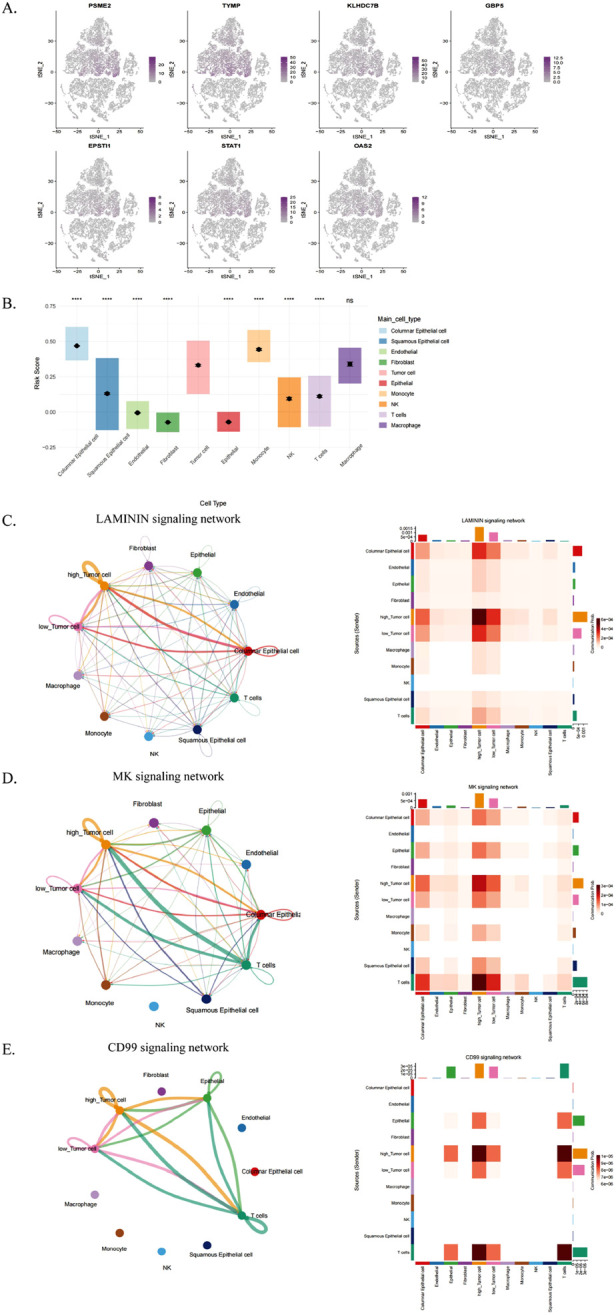
Analysis of TME differences between ICDR-based risk subgroups. **(A)** Single-cell RNA-seq analysis of PSME2, TYMP, KLHDC7B, GBP5, EPSTI1, STAT1, and OAS2 expression across various cell types. **(B)** ICDR scoring across cell types using tumor cells as a reference. **(C–E)** Interaction diagrams depicting cell communication networks for the LAMININ, MK, and CD99 signaling pathways among different cell types.

We analyzed the role of ICDRs in tumor immune microenvironment by using the previous data of single cell transcriptome, and we analyzed the distribution of several key genes (PSME2, TYMP, KLHDC7B, GBP5, EPSTI1, STAT1 and OAS2) in different cell types (Figure 9A). These genes are mainly distributed in columnar epithelial cells, tumor cells and monocytes. Subsequently, we use tumor cells as a standard to analyze and predict the risk scores of different cells in the model (Figure 9B). Columnar epithelial cells, monocytes and macrophages scored higher than tumor cells.

Cellular communication plays a role in tumour immunity through a variety of mechanisms, particularly in the interaction of malignant tumour cells with other cell subpopulations. Therefore, in this study, tumour cells were classified into high-risk and low-risk groups based on risk scores of ICDRs, their cellular communication patterns in the tumour microenvironment (TME) were analysed, and potential interactions with other cell types were assessed. The results showed that high-risk tumour cells communicated more actively with multiple cell types, especially in the LAMININ, MK and CD99 signalling pathways (Figures 9C–E). This phenomenon suggests that high-risk tumour cells may play a more important role in tumour progression, which may also explain their poorer prognosis.

### 3.9 Correlation analysis between ICDRs and immune infiltrating cells

**FIGURE 10:**
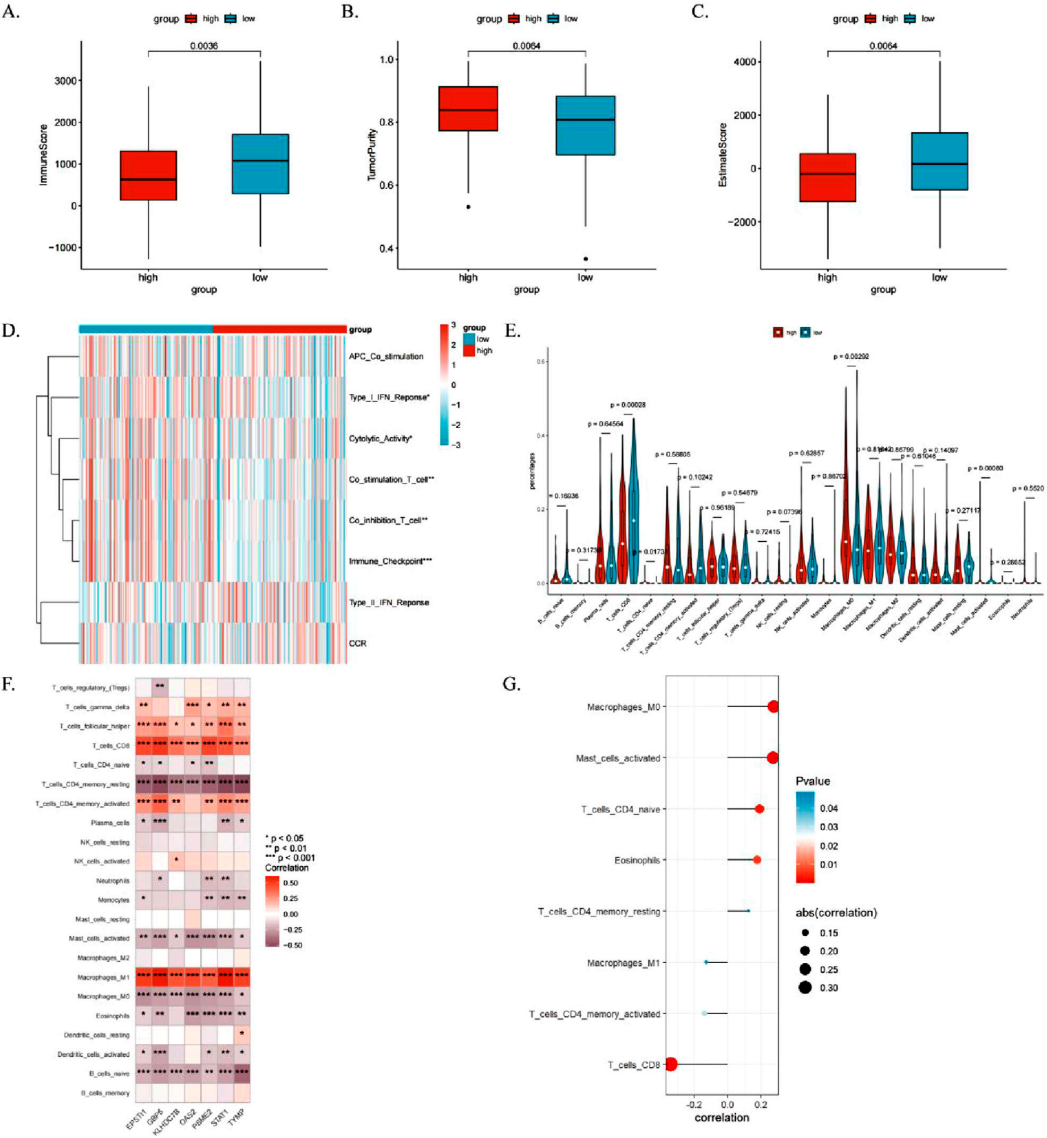
Relationship between ICDRs and immune cell infiltration in the immune microenvironment. **(A–C)** Comparison of immune status between high- and low-risk subgroups using immune scores, ESTIMATE scores, and tumor purity. **(D, E)** Analysis of the relationship between immune pathway activity and risk groups using the ssGSEA and CIBERSORT algorithms. **(F)** Heatmap showing associations between TME-infiltrating cells and ICDR-related genes. **(G)** Correlation analysis between TME-infiltrating cells and ICDR-related genes. *P < 0.05; **P < 0.01; ***P:< 0.001; ****P < 0.0001.

The immune microenvironment of CESC patients was comprehensively analyzed using multiple computational algorithms, including ESTIMATE, ssGSEA, and CIBERSORT. The analysis revealed that the high-risk group exhibited significantly higher tumor purity but lower immune and ESTIMATE scores (Figures 10A–C). Moreover, the high-risk group demonstrated elevated cytolytic activity and type II interferon response activity (Figure 10D). Immune cell infiltration profiling indicated a greater abundance of CD8^+^ T cells and M1 macrophages in the low-risk group, whereas CD4^+^ memory-activated T cells, M0 macrophages, and dendritic cells were more active in the high-risk group (Figure 10E; Supplementary Table S5).

The heat map shows the correlation between the seven genes and tumour-infiltrating immune cells in the ICDRs prediction model (Figure 10F). It is worth noting that CD4^+^ memory activated status, CD8 T cells and M1 macrophages are positively correlated with the seven genes, while M0 macrophages and activated plasma cells are significantly negatively correlated. Pearson correlation analysis identified eight immune cell types significantly associated with ICDRs (P < 0.05) (Figure 10G). Kaplan-Meier survival analysis revealed that six immune cell types—M2 macrophages, resting mast cells, activated NK cells, monocytes, resting NK cells, and M0 macrophages—were significantly correlated with patient prognosis (log-rank test, P < 0.05). These findings underscore the critical role of immune cell infiltration within the tumor microenvironment (TME) in influencing survival outcomes (Supplementary Figures S4A–F).

### 3.10 Analysis of the correlation between ICDRs and the anti-cancer immune cycle, as well as the response to immunotherapy

**FIGURE 11:**
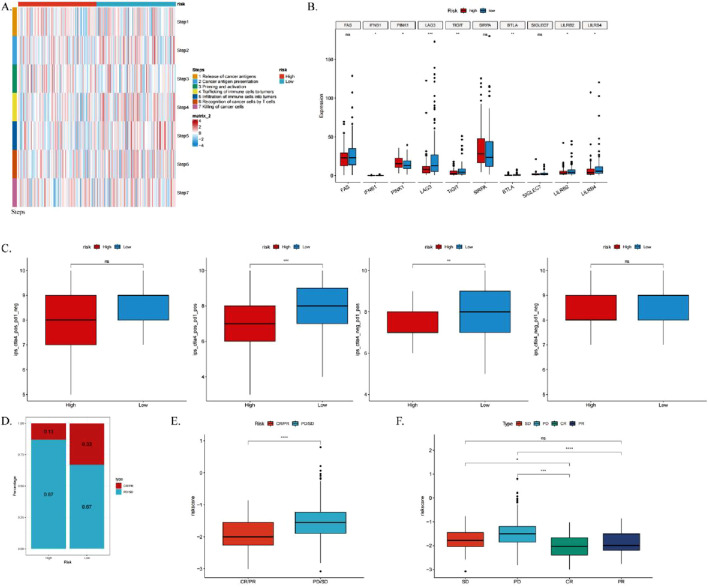
Analysis of the correlation between ICDRs and the anti-cancer immune cycle, as well as the response to immunotherapy. **(A)** Heatmap illustrating differences in seven-step anticancer immune cycle activity between high- and low-risk groups. **(B)** Differential expression of immune checkpoints across high- and low-risk subgroups. **(C)** Comparison of IPS scores between high- and low-risk groups. **(D–E)** Box plots showing the proportion of patients with CR/PR or SD/PD receiving immunotherapy in the IMvigor210 cohort, alongside differences in risk scores. **(F)** Box plots depicting risk scores among patients with CR, PR, SD, and PD in the IMvigor210 cohort. *p < 0.05; **p < 0.01; ***p < 0.001.

To further investigate the relationship between ICDRs and immunotherapy, we evaluated the activity of each step in the anti-cancer immune cycle, aiming to elucidate the role of immune cells in cancer immune responses. Our analysis (Figure 11A) revealed significant differences between ICDRS risk subgroups at steps 2, 4, 6, and 7 of the anti-cancer immune cycle. These findings suggest that in the low-risk group, immune cells exhibit stronger anti-cancer activity throughout the functional cycle. Previous studies have shown that high expression of immune checkpoint inhibitors (ICIs) is associated with improved anticancer response. To further explore this, we analyzed the expression levels of immune checkpoints in ICDRs risk subgroups, and several immune checkpoints, including LAG3, LILRB4, and TIGIT, were significantly more expressed in the low-risk group (Figure 11B). In addition, to validate these findings, we examined the immune phenotype score (IPS) in the TCIA database. Higher IPS scores generally predict better response to ICI treatment. Our analysis showed that the IPS score was higher in the low-risk group regardless of the expression pattern of PD-1 and CTLA-4, further supporting our hypothesis (Figure 11C).

Furthermore, to assess the clinical relevance of ICDRs in immunotherapy response, we analyzed the IMvigor210 cohort. We calculated the risk scores for each patient, categorizing them into high-risk and low-risk groups. Chi-square tests revealed that the low-risk group had a significantly higher proportion of patients with complete or partial responses (CR/PR), whereas the high-risk group exhibited a greater number of patients with stable disease or progressive disease (SD/PD) (Figure 11E). Moreover, CR/PR patients had significantly lower risk scores compared to SD/PD patients (Figures 11F, G), indicating that ICDRs play a crucial role in predicting immunotherapy outcomes and that patients in the low-risk group are more likely to experience favorable treatment responses. These findings collectively suggest that ICDRs serve as an important predictor of immunotherapy response, with patients in the low-risk group being associated with better clinical outcomes.

### 3.11 Analysis of the responsiveness to different chemotherapeutic agents in high-risk and low-risk groups

**FIGURE 12:**
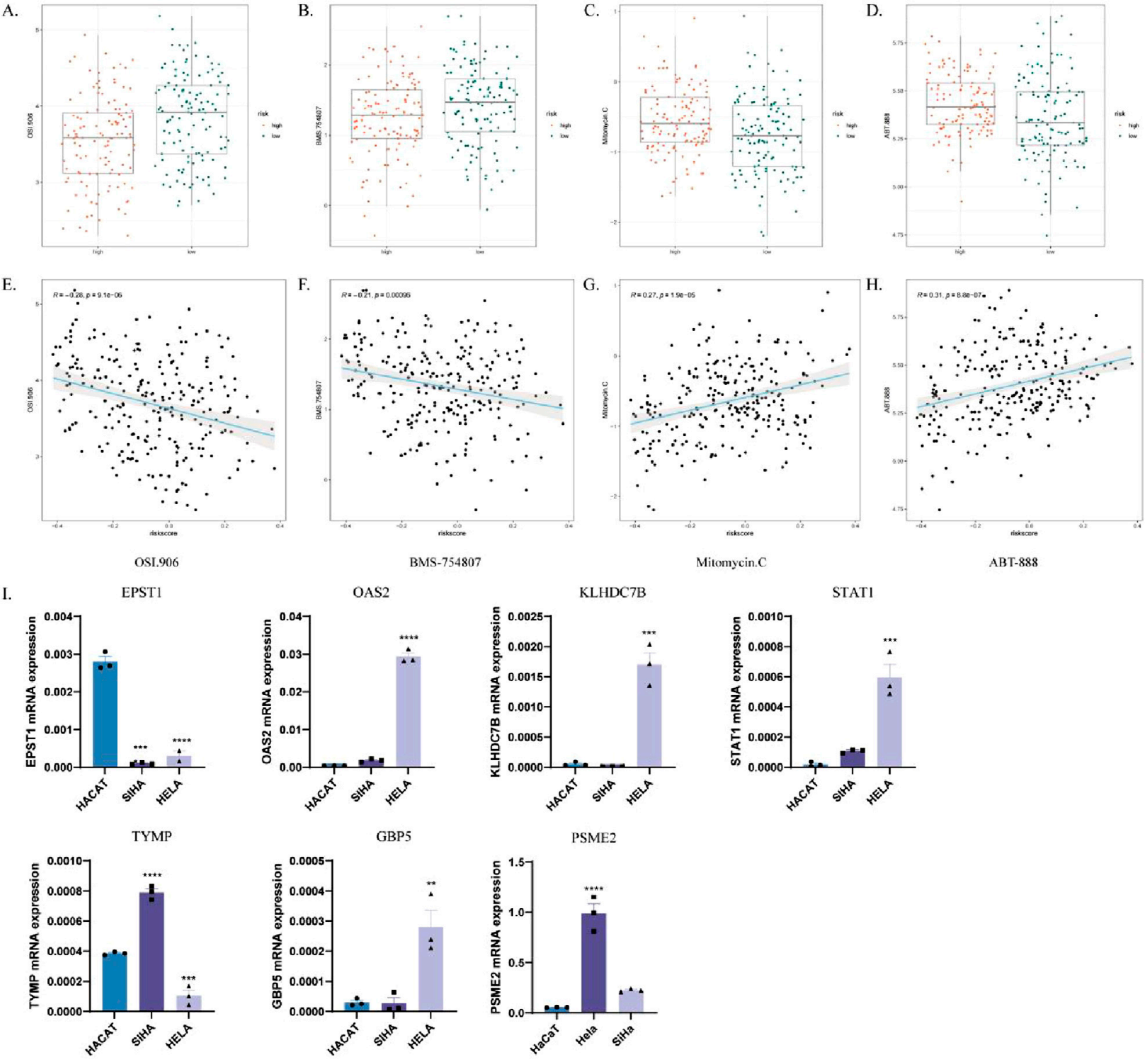
Analysis of the responsiveness to different chemotherapeutic agents in high-risk and low-risk groups. **(A–D)** Box plots comparing drug sensitivity to OSI-906 **(A)**, BMS-754807 **(B)**, mitomycin C **(C)**, and ABT-888 **(D)** in high- and low-risk groups. **(E–H)** Correlation analysis of IC50 values with risk scores for OSI-906 **(E)**, BMS-754807 **(F)**, mitomycin C **(G)** and ABT-888 **(H)**. **(I)** Verify the mRNA expression levels of the seven genes in the ICDRs prediction model in HaCaT, Hela and SiHa cells. *p < 0.05; **p < 0.01; ***p < 0.001.

We assessed the IC50 values of targeted therapies in ICDRs risk subgroups using tumor drug sensitivity genomics (GDSC). The results showed that OSI-906 and BMS-754807 exhibited lower IC50 values in the high-risk group, indicating greater sensitivity (Figures 12A, B). Correlation analysis revealed a significant negative correlation between risk score and IC50 for these drugs (Figures 12E, F). Conversely, sunitinib and ABT-888 had lower IC50 values in the low-risk group, with a significant positive correlation to the risk score (Figures 12C, D, G, H). These findings suggest that the high-risk group is more sensitive to OSI-906 and BMS-754807, while the low-risk group responds better to mitomycin C and ABT-888.

To validate the gene expression levels in the prediction model of ICDRs, the mRNA expression levels of the seven ICDR genes were analyzed in cervical cancer cell lines, including normal epithelial cells (HaCaT) and two cervical cancer cell lines (SiHa and HeLa). The analysis showed that OAS2, KLHDC7B, STAT1, TYMP, PSME2 and GBP5 were significantly upregulated in cervical cancer cells compared with normal epithelial cells (Figures 12I–N).

## 4 Discussion

Cervical cancer is one of the most common gynecological tumors, with about 500,000 new cases diagnosed globally each year, including about 90,000 cases in China (Malagón et al., 2024). At present, the incidence of cervical cancer continues to rise, and the age of onset gradually tends to be younger. Although the HPV vaccine has been widely promoted, cervical cancer remains a major global health challenge (Liu K. et al., 2024). This situation highlights the urgent need for precision medicine to promote early detection, timely diagnosis, and effective treatment (Desravines et al., 2024). Immunotherapy, as the most promising tumor treatment method, plays a key role in determining patient prognosis and treatment strategy (Chen et al., 2024; Liu et al., 2024c). Nevertheless, challenges such as low immunogenicity, gene instability, and antigenic variation remain, causing many tumor cells to escape immune surveillance (Han et al., 2024). Many studies have focused on finding new targets to optimize treatment strategies and improve patient outcomes, These included genes related to angiogenesis (Deng et al., 2024), matrix immune marker genes (MIS) (Czekay et al., 2022), and anoikis-related genes (ARGs) (Wang M. et al., 2024). However, although immunogenic cell death (ICD) has been suggested to have the ability to induce specific immune responses and regulate tumor immunity (Wang et al., 2018), its role in cervical cancer has not been fully focused. In this study, by identifying immunogenic genes associated with cervical cancer, a variety of machine learning algorithms were used to construct an ICDRs prognostic model, and the relationship between ICDRs and immune infiltrating microenvironment was further analyzed.

In this study, we analysed immune-related genes associated with CESC using single-cell datasets, combined WCGNA and RNA-seq analyses, and constructed a prediction model for ICDRs using a multi-machine algorithm. The prediction model for ICDRs was found to exhibit robust predictive performance by validation in multiple CESC-related cohorts. Further univariate and multivariate Cox regression analyses showed that the prediction model for ICDRs had the potential to be an independent prognostic indicator. Subsequent analysis of clinical indicators of CESC patients using the ICDRs prediction model found a significant correlation between patient survival status, M and N staging and the ICDRs prediction model, further highlighting its potential to more accurately monitor tumour progression and prognosis by integrating molecular and clinical pathological indicators, surpassing traditional TNM staging. Modern research has shown that in targeted therapy for triple-negative breast cancer, restoring IFN-γ signalling *in vivo* can enhance the immune response (Lazovic et al., 2025). In PD-1 immunotherapy, IFN-γ signalling can be activated by inhibiting glycolysis and restoring the activity of the HK2/Lactate/IFN-γ axis, thereby further enhancing the immune response (Xu et al., 2025). In addition, the recombinant HPV-16E7d vaccine can also promote the immune response by activating IFN-γ, thereby achieving an anti-tumour effect (Gachpazan et al., 2025). The significant enrichment of immune-related pathways, especially those related to IFN-γ and various cytokines, in the low-risk group further indicates that patients in this group may have a better prognosis, which may be closely related to enhanced immune activation and a stronger immune response. On the other hand, studies have also shown that inhibiting epithelial-mesenchymal transition (EMT) can effectively resist tumor drug resistance and metastasis, while inhibiting glycolysis is considered a potential strategy for cancer treatment (Ma et al., 2023; Mao et al., 2025). Our study suggests that the high-risk group may have a more aggressive phenotype and a poorer prognosis. This immune response helps to stop tumour growth by enhancing the ability of the immune system to detect and clear tumour cells. These findings highlight the importance of tailoring interventions to the different characteristics of high- and low-risk groups in order to effectively control the progression of cervical cancer.

In the tumour immune microenvironment (TME), complex intercellular communication between tumour cells, immune cells and stromal cells plays a crucial role in tumour progression and treatment response (Yin et al., 2024). Our analysis identified the mutual communication between different cell subsets, and the results showed that despite the existence of cell communication, there was a break in most of the intercellular communication in the high tumour cell group. This break may lead to a lack of communication of immune cells, which in turn helps tumour cells escape from the surveillance and attack of the immune system. In addition, we also analysed the relationship between ICDRs and the TME, and the results showed that the low-risk group had significantly higher scores in terms of stromal score and immune score. The low-risk group was significantly positively correlated with TME-related characteristics, including immune checkpoints and type I interferon responses. Of particular note, the low-risk group showed a significant increase in CD8^+^ T cells and M1 macrophages, which have been shown to promote the infiltration of inflammatory cells in the tumour microenvironment (Chen et al., 2023), further demonstrating that patients in this group have a better prognosis through a stronger immune response. In addition, it was found that seven genes in the ICDRs prediction model were positively correlated with CD4^+^ memory activated status, CD8 T cells and M1 macrophages. This phenomenon may reveal that the genes related to the ICDRs prediction model can regulate the active participation of immune cells and promote the immune clearance of tumors.

Genetic mutations play an important role in the occurrence, progression, treatment response and recurrence of tumours (Moreno-Gonzalez et al., 2024; Yin et al., 2024). We analysed the genetic mutation characteristics and intratumour heterogeneity (ITH) of different risk subgroups, and the results showed that the high-risk subgroup had a higher ITH level and enhanced metastasis potential, which was consistent with its poorer prognosis. This was further supported by the validation results of the IMvigor210 cohort, which showed that patients in the low-risk group had higher complete response (CR) and partial response (PR) rates, while patients with stable disease or progressive disease (SD/PD) had higher risk scores, indicating a poorer prognosis for the high-risk group. These results indicate that the ICDRs risk score can effectively predict the outcome of immunotherapy, and that low-risk patients may obtain more significant treatment benefits. In addition, drug sensitivity analysis revealed four potentially effective therapeutic drugs: mitomycin C, ABT-888, BMS-754807, and OSI-906, which can significantly inhibit tumour progression in the clinic. Mitomycin C inhibits tumour growth by targeting DNA synthesis (Gorodnova et al., 2020). OSI-906 and BMS-754807 are IGF-1R/IR inhibitors (Godina et al., 2024), the former of which affects tumour progression by regulating the insulin and IGF-2 signalling pathways influences tumour progression (Lodjak et al., 2023); the latter induces apoptosis and shows significant anti-tumour effects by inhibiting the ATP-competitive tyrosine kinase activity of IGF-1R (Hassan et al., 2024). ABT-888, a PARP inhibitor, enhances anti-tumour immune responses by regulating DNA repair mechanisms (Li et al., 2023; Luo et al., 2024). These drugs have good application prospects in the immunotherapy of cervical cancer.

The seven key genes in the ICDRs prediction model play an important role in the progression of various tumors and have broad clinical application prospects as potential biomarkers. For example, EPST1 is considered to have immunomodulatory activity and show anti-tumor potential (Zhang X. et al., 2024); OAS2, as a T cell exhaustion-related gene, has been shown to play a role in the regulation of immune responses in breast cancer and non-small cell lung cancer (Lu et al., 2024; Ching et al., 2025). In addition, KLHDC7B has been found to promote the proliferation and migration of bladder urothelial carcinoma cells (Hou et al., 2024). STAT1 plays a key role in regulating the effect of chemotherapy (Buttarelli et al., 2019); TYMP is considered to be a potential prognostic marker for ovarian cancer and breast cancer (Liu S. et al., 2025; Tao et al., 2025); GBP5, as an immune response regulator, can affect tumor progression (Zou et al., 2024); and PSME2 is closely related to the progression of various tumors (Li R. et al., 2024). The above analysis shows the important biological functions of the relevant genes in the ICDRs prediction model in different tumor types, and further confirms the broad application prospects of the prediction model in prognosis assessment. Through further experiments, we verified the expression levels of the seven genes in the ICDRs prediction model in cervical squamous cell carcinoma and found that most of the genes were overexpressed. Overall, these results further support the reliability of risk scores as predictive indicators.

Translating ICDRs predictive models into clinical practice is of great significance in helping patients and clinicians make more accurate and personalised treatment decisions. First, the models provide personalised treatment plans for patients, so that they can make more accurate treatment choices based on specific risk scores. Patients in the high-risk group need to receive more active treatment interventions, while patients in the low-risk group can adopt standardised treatment plans to optimise treatment effects. Second, predictive models help optimise the intensity of treatment and the allocation of medical resources. In clinical practice, patients in the high-risk group require more attention and intensive treatment, while patients in the low-risk group can avoid over-treatment, reduce the burden on medical care, and improve overall medical efficiency. Third, ICDRs prediction models can improve the effectiveness of early intervention and disease monitoring. Compared with patients in the low-risk group, patients in the high-risk group should be more actively intervened at an early stage, so as to improve treatment effects, prolong survival, and reduce misdiagnosis. Fourth, the model promotes shared decision-making. By providing patients with clear risk information, they are fully informed and can participate in clinical decision-making, making informed choices based on personal risk, thereby improving treatment compliance. Fifth, ICDRs predictive models provide important support for long-term treatment outcome evaluation and follow-up research. As the model continues to be optimised and validated, it provides a new perspective on clinical treatment and helps to promote the exploration of new treatment options.

In summary, this study constructed a risk prediction model based on seven ICDRs genes through multi-omics joint analysis. The model can effectively predict patient prognosis, tumour microenvironment (TME) characteristics, immune function, immunotherapy response and drug sensitivity. This study combined machine learning algorithms and single-cell transcriptome technology to construct an ICDRs prediction model, providing new ideas for personalised treatment strategies for cervical cancer (CESC) patients. Current research has shown that ICDRs are closely related to tumorigenesis and development in multiple tumor types, such as hepatocellular carcinoma (Sun et al., 2024), colorectal cancer (Liu H. et al., 2025), ovarian cancer (Gao et al., 2024), and lung adenocarcinoma (Li S. et al., 2024). However, the role of ICDRs in other tumor types needs to be further verified and studied in depth. In the context of prediction, prevention and personalised medicine (PPPM), our findings can guide the development of new therapies and prevention strategies for cervical cancer (Wang et al., 2018). By improving disease management and reducing economic burden, ICDRs prediction models are expected to be an important tool for advancing precision medicine for cervical cancer.

## 5 Conclusion

In this study, we constructed an ICDRs model to predict the prognosis of cervical cancer patients and revealed immune differences between different risk groups, highlighting its potential for clinical application. In addition, we predicted the sensitivity of patients to immunotherapy and chemotherapy, providing a valuable reference for personalised treatment. Overall, the application of this model in clinical practice can effectively improve the prognosis assessment of cervical cancer patients, optimise treatment decisions, and provide more accurate personalised treatment plans.

## Data Availability

The datasets presented in this study can be found in online repositories. The names of the repository/repositories and accession number(s) can be found in the article/Supplementary Material.
