# Supplementary material for: Immunogenic cell death genes in single-cell and transcriptome analyses perspectives from a prognostic model of cervical cancer
Source: Front Genet. 2025 Apr 7;16:1532523. doi: 10.3389/fgene.2025.1532523 (PMC12009919; doi:10.3389/fgene.2025.1532523)
Supplement: Supplementary file 1 [file Supplementaryfile1.docx]

Supplementary Material

Supplementary Figures


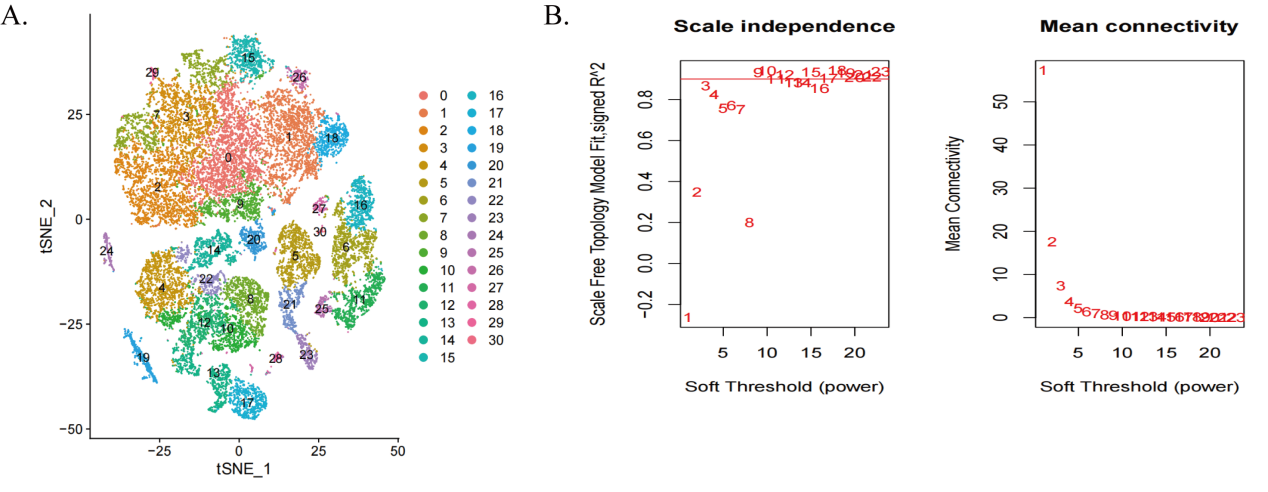
**Supplementary Figure 1**. (A) t-SNE plots coloured by different cell populations; (B) Determination of the optimal soft threshold for WGCNA analysis.


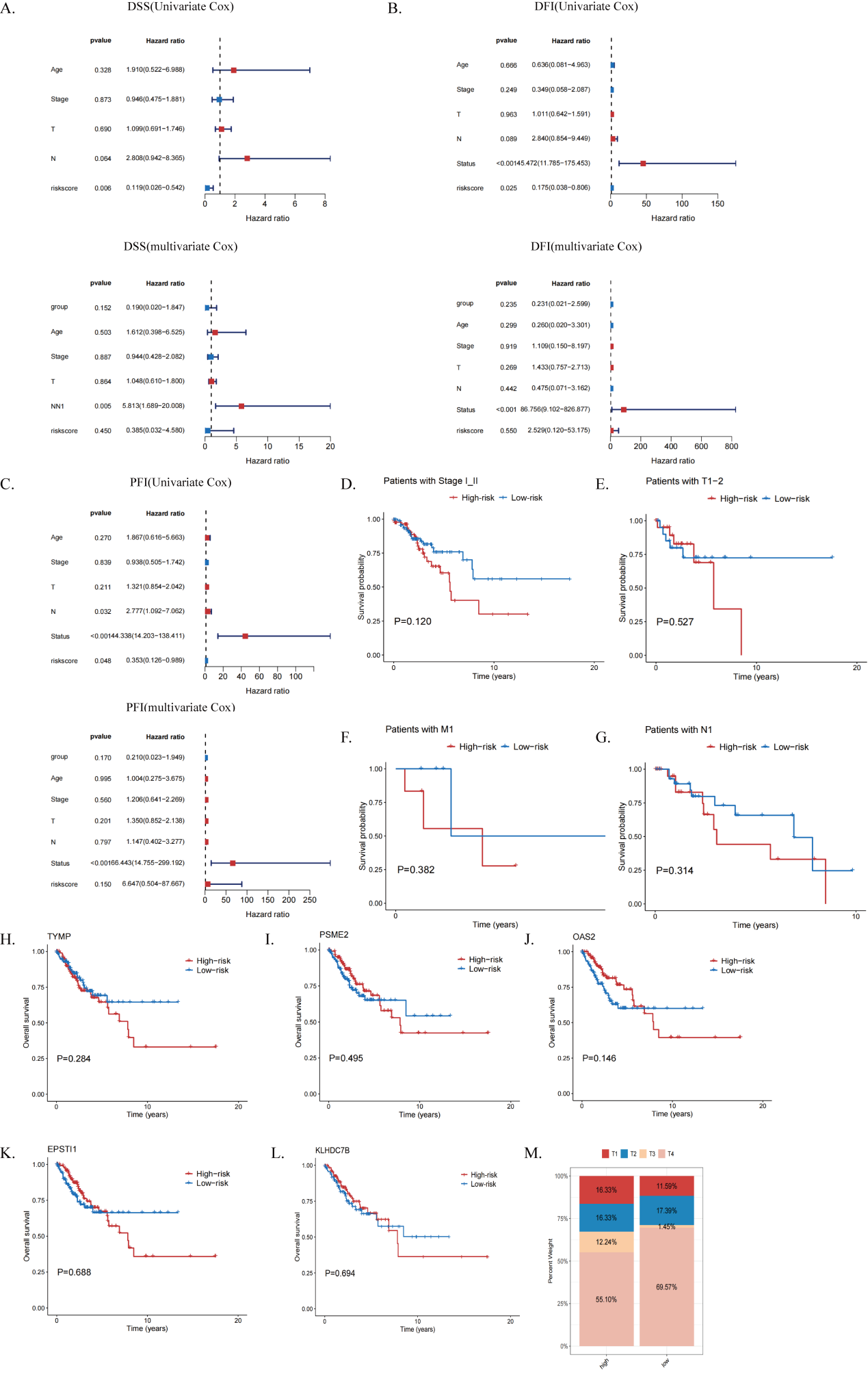
**Supplementary Figure 2**. (A-C) Univariate and multivariate Cox analyses between DSS; DFI; PFI and clinical characteristics in TCGA-CESC; (D-G) KM curves showing survival analyses of ICDRS in risk subgroups of CESC patients; including StageI-II; T1-2; M1; N1; (H-I) KM survival curves for the ICDRs gene including TYMP(H); PSM2(I); OAS2(J); EPSTI1(K); KLHDC7B(I); (M) The proportion of T stage in ICDRs risk subgroups.

**
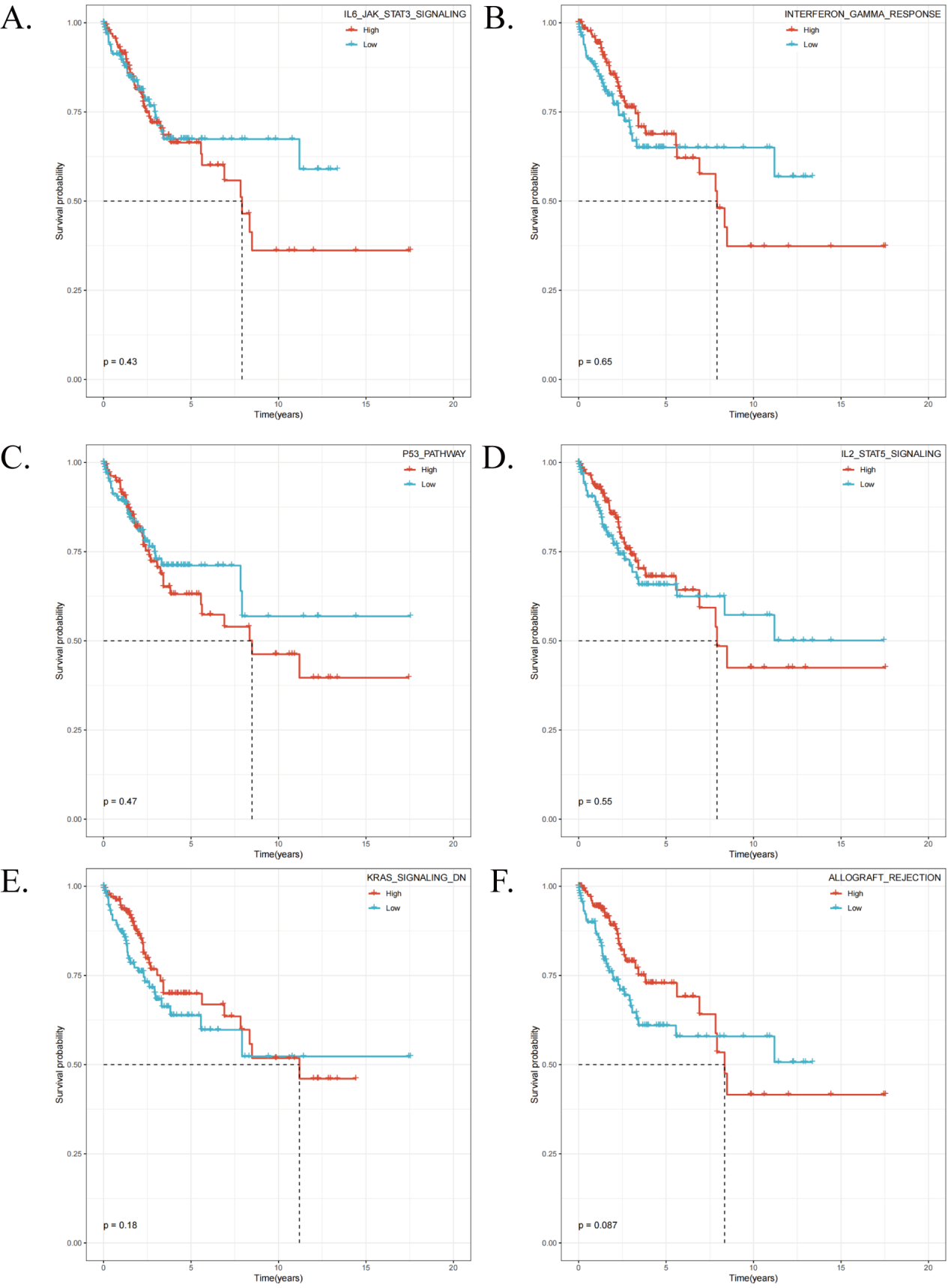
**

**Supplementary Figure 3. (**A-F) Kaplan-Meier (KM) analysis was performed on pathways including IL-6_JAK_STAT3_SIGNALING(A); INFLAMMATORY_RESPONSE(B);P53_PATHWAY(C); IL2_STAT5_SIGNALING(D); KRAS_SIGNALING_DN(E); and ALLOGRAFT_REJECTION(F).


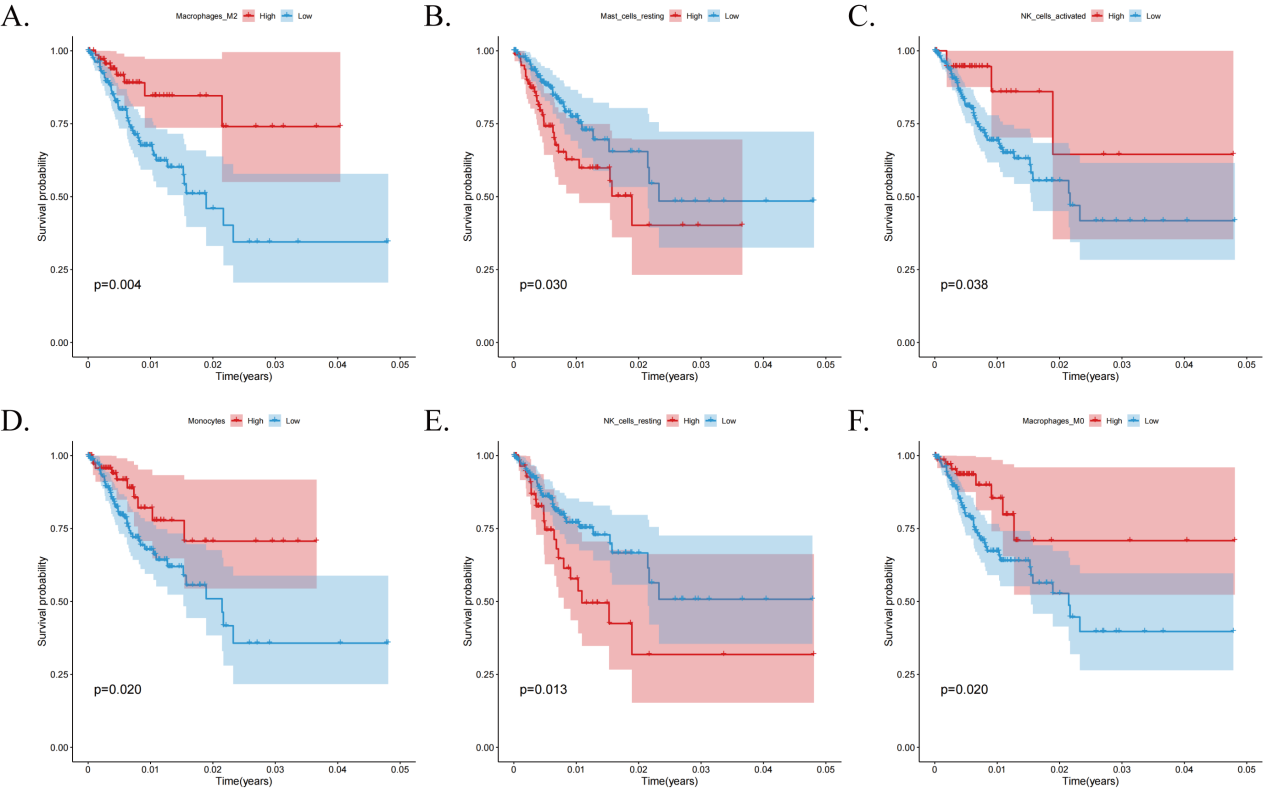
**Supplementary Figure 4. (**A-F) Comparison of immune cell populations between high- and low-risk groups; as calculated by the CIBERSORT algorithm; including resting dendritic cells (A); M2 macrophages (B); resting mast cells (C); activated NK cells (D); monocytes (E); resting NK cells (F); and M0 macrophages (G).
